# *Beta vulgaris* as a Natural Nitrate Source for Meat Products: A Review

**DOI:** 10.3390/foods10092094

**Published:** 2021-09-04

**Authors:** Paulo E. S. Munekata, Mirian Pateiro, Rubén Domínguez, Marise A. R. Pollonio, Néstor Sepúlveda, Silvina Cecilia Andres, Jorge Reyes, Eva María Santos, José M. Lorenzo

**Affiliations:** 1 Centro Tecnológico de la Carne de Galicia, Rúa Galicia No. 4, Parque Tecnológico de Galicia, San Cibrao das Viñas, 32900 Ourense, Spain; mirianpateiro@ceteca.net (M.P.); rubendominguez@ceteca.net (R.D.); jmlorenzo@ceteca.net (J.M.L.); 2 Department of Food Technology, School of Food Engineering, State University of Campinas (Unicamp), Campinas 13083-862, SP, Brazil; pollonio@unicamp.br; 3 Departamento de Producción Agropecuaria, Facultad de Ciencias Agropecuarias y Forestales, Universidad de La Frontera, Campus Integrado Andrés Bello Montevideo s/n, Temuco 4813067, Chile; nestor.sepulveda@ufrontera.cl; 4 Centro de Investigación y Desarrollo en Criotecnología de Alimentos (CIDCA), Consejo Nacional de Investigaciones Cientificas y Tecnicas (CONICET), Facultad de Ciencias Exactas, Universidad Nacional de La Plata, CIC-PBA, 47 y 116, La Plata 1900, Argentina; scandres@biol.unlp.edu.ar; 5 Departamento de Ciencias Agropecuarias y Alimentos, Universidad Técnica Particular de Loja, Calle París, San Cayetano Alto, Loja 110107, Ecuador; freyes@utpl.edu.ec; 6 Area Academica de Quimica, Universidad Autonoma del Estado de Hidalgo, Carr. Pachuca-Tulancingo Km. 4.5, Mineral de la Reforma, Hidalgo 42184, Mexico; emsantos@uaeh.edu.mx; 7 Área de Tecnología de los Alimentos, Facultad de Ciencias de Ourense, Universidad de Vigo, 32004 Ourense, Spain

**Keywords:** cured meat products, beetroot, chard, spinach beet, nitrite, starter culture

## Abstract

Curing meat products is an ancient strategy to preserve muscle foods for long periods. Nowadays, cured meat products are widely produced using nitrate and nitrite salts. However, the growing of the clean-label movement has been pushing to replace synthetic nitrate/nitrite salts (indicated as E-numbers in food labels) with natural ingredients in the formulation of processed foods. Although no ideal synthetic nitrate/nitrite replacements have yet been found, it is known that certain vegetables contain relevant amounts of nitrate. *Beta vulgaris* varieties (Swiss chard/chard, beetroot, and spinach beet, for instance) are widely produced for human consumption and have relevant amounts of nitrate that could be explored as a natural ingredient in cured meat product processing. Thus, this paper provides an overview of the main nitrate sources among *Beta vulgaris* varieties and the strategic use of their liquid and powder extracts in the production of cured meat products.

## 1. Introduction

Curing is an old meat preservation strategy that consists of the use of marine salt containing nitrate on the surface of meat cuts and pieces [1]. Currently, it is known that the curing occurs mainly from the action of nitrate (NO_3_^−^) and nitrite (NO_2_^−^) that improve color stability, slow oxidative reactions, and impart the characteristic cured flavor to meat products [2]. The process is centered in the stabilization of the iron atom in the porphyrin ring of myoglobin. Nitric oxide (obtained from nitrite) interacts with the iron atom of myoglobin and generates nitrosocompounds, which increases the structural stability of this muscle pigment by preventing the loss of iron [3].

Once the iron atom of myoglobin is preserved in the structure of myoglobin, the oxidative stability of the meat product improves and the progression of oxidative reactions catalyzed is limited. Consequently, the degradation of lipids and proteins as well as sensory decay during storage are delayed [4,5]. Nitrite also improves the safety of meat products by inhibiting the growth of spoilage and pathogenic microorganisms, particularly *Clostridium botulinum*, which produces one of the most potent lethal neurotoxins [6].

Replacing nitrate and nitrite salts in meat product processing is a major challenge for researchers and professionals in the meat industry. Due to its multifunctional effect on quality, safety, and shelf life, an ideal replacer has not yet been found [3]. Another main factor considered in this context is the growing interest among consumers to avoid the consumption of food products with unfamiliar or synthetic additives, such as those indicated with E-numbers (E 249, E 250, E 251, and E 252 for sodium and potassium nitrate and potassium and sodium nitrite, respectively) [7]. Consequently, the movement known as the clean-label movement has emerged among consumers who are interested in the consumption of food products with a healthiness appeal [8,9,10,11,12]. The concept and definition of “clean” has not been comprehensively defined and may contain the expressions “natural,” “based on,” and “free from,” depending on the food and the ingredient, for instance [13]. Moreover, the consumers’ understanding about food characteristics, the technical importance of ingredients and additives, the perception of health risk, exposure to media, and price are other factors that are known to influence the perception of healthiness among consumers [7]. It is also important to note that consumers may categorize food additives into two groups: ingredients/additives perceived as “known-natural-good” and those perceived as the opposite of each one of these concepts, which leads to either the acceptance or the rejection of the ingredient/food, respectively [14].

In this sense, the use of natural extracts rich in technologically relevant compounds has been proposed for the production of cured muscle foods [10,15,16]. Vegetables are exceptional sources of technologically relevant compounds that have been widely studied in food processing. Among the potential candidates, *Beta vulgaris* stands out due to the high nitrate content and wide production in all continents. This vegetable family includes the common beetroot (*Beta vulgaris* subsp. *vulgaris* var. *vulgaris*), sugar beet (*Beta vulgaris* subsp. *vulgaris* var. *altissima*), chard/Swiss chard (*Beta vulgaris* subsp. *vulgaris* var. *cicla*), and spinach beet (*Beta vulgaris* var. *bengalensis*), for instance [17,18].

Due to the absence of a review describing the use and effect of *Beta vulgaris* extracts as natural curing agents, this paper aims to provide an overview of the nitrate content of *Beta vulgaris* varieties and their role as natural curing agents in the production of cured meat products.

## 2. Nitrate Content in *Beta vulgaris* Varieties

The recent studies reporting the content of nitrate in some subspecies of *Beta vulgaris* are presented in Table 1. Comparatively, most of these studies evaluated the content in chard, especially commercial samples. The content of nitrate observed in these studies varied from very low (<200 mg/kg) and low (200–500 mg/kg) to extremely high (>5000 mg/kg) [19,20,21,22,23,24,25,26,27,28,29,30,31,32,33]. Only the study carried out by Menal-Puey and Asensio [29] reported values in the range of medium (500–1000 mg/kg) and extremely high. Other interesting aspects of the recent studies presented in Table 1 are the influence of the fertilizing level, the season, the geographical area, and the production in the hydroponic system.

The level of fertilizers applied to the soil to produce chard can influence the content of nitrate in this vegetable [30]. According to these authors, increasing the level of fertilizers (either the commercial product or ammonium nitrate) has a significant effect on the nitrate content of both petioles and blades of this vegetable. Moreover, a tissue-dependent effect was also indicated by the authors, who reported a higher nitrate content of petioles than in blades, regardless of the fertilizer and dosage. Differently, the study carried out by Liu et al. [20] did not find significant differences in the nitrate content of chard leaves due to potassium deficiency or sodium supplementation in the soil. An interesting outcome related to the accumulation of nitrate in the leaves of chard is the growth using the hydroponic system. For instance, Bulgari et al. [28] reported an accumulation of 1061 mg/kg in chard leaves. Likewise, Kaburagi et al. [26] indicated an intense accumulation of nitrate in the leaves of chard produced with different levels of saline fish wastewater.

Brkić et al. [23] reported significant differences among chard samples in Croatia due to the season in Zagreb (1049.4 *vs.* 2260.2 mg/kg in spring and fall, respectively). However, non-significant differences were reported for samples collected from Osijek, Rijeka, and Split areas (values ranging from 248.5 to 2260.2 mg/kg for samples collected in Split and in Zagreb, both during the fall, respectively). Another study indicated that variations in chard samples collected from a single location can occur [22]. In this case, the samples collected in Canary Islands (Spanish archipelago in the Atlantic Ocean) had a nitrate content of up to 4362.2 mg/kg. According to the authors, this range of variation is related to the natural high capacity of nitrate accumulation in chard and the agricultural practices, especially with the use of nitrogen for cultivation of this vegetable.

The effect of seasons and the consequent differences in the exposure to sunlight have been indicated as factors that may influence the content of nitrate in chard [21]. This consideration was indicated to explain the variations observed among samples collected between 2009 and 2013 in Valencia (Spain). Similarly, Kyriacou et al. [31] argued that sunlight can affect nitrate reductase activity and increase the accumulation of nitrate in chard during periods of low sun incidence (fall and winter, for instance).

The nitrate content in the roots of beetroots also shows variations among studies carried out with samples purchased in markets [34,35,36,37,38,39,40]. The quantity reported in these studies varied from very low to very high (2500–5000 mg/kg). It is interesting to note that nitrate also accumulated in other beetroot tissues, such as the leaf, wherein nitrate values ranged from very low to very high in the leaf lamina and petiole [34].

A similar scenario can be observed in studies aiming to quantify the content of nitrate in the leaves of spinach beet [41,42]. In these studies, the nitrate content was between low and high levels. Another member of this group is sea beet (*Beta vulgaris* (L.) subsp. *maritima* (L.) Arcang.), which shows medium nitrate levels [43]. Considering the recent scientific evidence included in this review and the potential application of *Beta vulgaris* varieties as natural sources of nitrate for the production of meat products, the use of chard produced in the hydroponic system seems the most relevant option.

## 3. Application of *Beta vulgaris* in Meat Products

The use of natural extracts of *Beta vulgaris* in meat products is shown in Table 2. Three main strategies have been applied: direct addition of extracts, addition of extracts with pre-converted nitrite (pre-fermented extracts), and the combined use of nitrate-rich extracts with starter cultures.

### 3.1. Direct Addition of Beta vulgaris Extracts in Meat Products

Regarding the first strategy, the direct application of *Beta vulgaris* extracts (as sources of nitrate) with nitrite salts was explored in the study carried out by Jin et al. [45]. The authors reported a significant increase in redness and sensory acceptance of color in cooked pork sausages produced with powdered beetroot extract but no significant effect in terms of lipid oxidation. Other sensory attributes and texture were not affected by the extracts. In a related experiment with cooked pork sausage, a mixture of natural extracts containing powdered beetroot extract (also containing pomegranate, lemon, and rosemary extracts) did not affect lipid oxidation, volatile basic nitrogen (VBN), and sensory acceptance of color during storage [46]. In this case, the sensory properties (except for color) and color parameters were enhanced.

In another study on fresh beef sausage, powdered beetroot extract reduced the growth of coagulase-positive *Staphylococcus* when combined with a low concentration of thyme essential oil (0.0095%) for 28 days at 4 °C [47]. Moreover, a significant increase in sensory properties (appearance, color, and flavor) was also reported. In general, the incorporation of beetroot extracts in sausages without any pre-treatment or combination with a starter culture seems to be limited to effects on color [45,46,47]. This hypothesis is supported by the absence of major effects in terms of lipid oxidation [45,46] or microbial growth inhibition [46,47] during storage. In this sense, it is important to consider alternative strategies to convert nitrate to nitrite.

### 3.2. Fermentation of Beta vulgaris to Produce Nitrite-Rich Extracts

**Figure 1:**
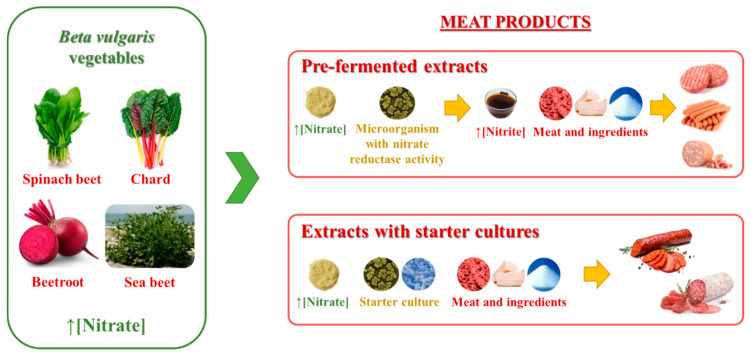
Schematic representation of the main strategic uses of *Beta vulgaris* extracts in the production of cured meat products.

An interesting strategy to explore the natural formation of nitrite is by fermenting nitrate-rich extracts by either fermenting the extracts before the incorporation into the meat product formulation or adding the extracts with a starter culture to the formulation of the meat product (Figure 1) [15,48,49].

Regarding the second strategy, the use of *Staphylococcus carnosus* has been explored by many authors due to the relative mild fermenting conditions (30–37 °C for 24 h) to produce extracts rich in nitrite from both beetroot and chard (from 322 to 60,540 ppm; Table 2). In terms of application to meat products, the use of beetroot seems to be limited to synthetic nitrite. According to the experiment carry out by Hwang et al. [50], the incorporation of beetroot extracts rich in nitrite (3%) into pork sausage reduces redness and causes a significant increase in both thiobarbituric-acid-reactive substances (TBARS) and VBN values in relation to controls with synthetic nitrite. No effects were reported for L* and the total plate count between sausages produced with natural and synthetic nitrite. 

A related experiment with higher concentrations of beetroot extracts (at 5% and 10%) indicated a similar outcome in cooked pork sausage [51]. A significant reduction in the a* value and scores for sensory evaluation of color was obtained from sausages produced with this natural extract in comparison with control samples with synthetic nitrite. Additionally, TBARS values in samples prepared with beetroot extracts were higher than those reported for controls with synthetic nitrite.

Differently, the use of these nitrite-rich extracts from *Beta vulgaris* varieties can improve the shelf life of meat products. For instance, the study performed by Hwang et al. [52] compared the effects of beetroot extracts (1%, 3%, and 5%) on the stability of low-salt frankfurters during 20 days of refrigerated storage. These authors indicated that natural extracts (especially at 5%) improve the redness and reduce the formation of lipid oxidation products and VBN during storage. Similar scores for sensory appearance, color, juiciness, and overall acceptance were reported between controls with nitrite and treatments with different levels of beetroot extracts. 

A related experiment with chard fermented extracts (either 2 g extract/100 g or 1 g extract/100 g with 0.006% synthetic nitrite) indicated a similar capacity to preserve redness, protect lipids from oxidation, and preserve the sensory quality (color, flavor, off-flavor, tenderness, juiciness, and overall acceptability) of pork patties after 28 days at 4 °C [53].

**Table 2:** Effect of *Beta vulgaris* extracts rich in nitrate/nitrite on the quality and storage stability of meat products.

Source	Meat Product	Treatments and Nitrite Content in Extracts	Sampling Point	Residual Nitrate/Nitrite	Effect	Ref.
Beetroot	Cooked pork sausage	0.5% and 1.0% powder extract	Stored at 4 °C for 28 days	4.4–5.1 ppm	Reduced L* value; no effect on b* value, texture, TBARS, sensory scores for flavor, tenderness, juiciness, and overall acceptability; increased a* value and sensory score for color	[45]
Beetroot and other natural extracts	Cooked pork sausage	0.6% (1% beetroot powder in the mixed extract)	Stored at 4 °C for 4 weeks	0.6 ppm	No effect on pH, TBARS, VBN, microbial count, sensory score for color; increased L*, a*, and b* values, shear force, and sensory scores for aroma, flavor, juiciness, chewiness, and overall acceptability	[46]
Beetroot and thyme essential oil	Fresh beef sausage	1% powder extract	Stored at 4 °C for 28 days	n.e.	Reduced coagulase-positive *Staphylococcus* growth; no effect on sensory scores for odor, texture, and overall acceptability; increased aerobic mesophilic bacteria and sensory scores for appearance, color, and flavor	[47]
Beetroot	Cooked pork sausage	3% liquid extract (fermented with *Staphylococcus carnosus* at 30 °C for 24 h; 748 ppm nitrite)	Final product	~5 mg/kg	Reduced pH, a* value, residual nitrite; no effect on L* value and TPC; increased b* value, VBN, and TBARS	[50]
Beetroot	Cooked pork sausage	5% and 10% liquid extract (fermented with *Staphylococcus carnosus* at 30 °C for 24 h; 730 ppm nitrite)	Final product	15–30 mg/kg	Reduced pH, L*, and a* values, VBN, residual nitrite, and color scores; no effect on microbial counts, flavor, off-odor, and juiciness; increased b* value, TBARS, and overall acceptability (10%)	[51]
Beetroot	Low-salt frankfurters	1%, 3%, and 5% liquid extract (fermented with *Staphylococcus carnosus* at 30 °C for 24 h; 729 ppm nitrite)	Refrigerated storage for 20 days	n.e.	Reduced VBN, TBARS, TPC, L*, and b* values and tenderness; no effect on sensory appearance, color, juiciness, and overall acceptance; increased pH, a* value, and flavor	[52]
Chard	Pork patties	1 (with 0.006% synthetic nitrite) and 2 g powder/100 g (fermented with *Staphylococcus carnosus* at 37 °C for 24 h; 60,540 ppm nitrite)	Stored at 4 °C for 28 days	21–~60 mg/kg	Reduced pH and residual nitrite; similar TBARS, curing efficiency, redness preservation, and sensory scores as controls with nitrite	[53]
Beetroot	Fermented and dry-cured pork sausage	0.5% and 1% beetroot powder; *Staphylococcus carnosus* as starter culture	Ripening at 25 °C with RH of 95% for 1 day and decreasing 1 °C and 2% in RH every day for 6 days and at 15 °C with RH 75% for 27 days	0–209 mg nitrate/kg; 0–7.8 mg nitrite/kg	Reduced aw, pH (1%), L*, and b* values, residual nitrate and nitrite (0.5%), and lipid oxidation; no major effect on TPC, LAB, and total coliforms; increased weight loss, a* value, and formation of nitroso pigments	[54]
Beetroot	Fermented and dry-cured pork sausage	0.5% and 1% beetroot powder; *Staphylococcus carnosus* as starter culture	Stored at 5 °C for 60 days	0 mg nitrate/kg; 0–4.2 mg nitrite/kg	Reduced aw, pH, L*, and b* values, residual nitrate and nitrite, and nitroso pigments; no major effect on lipid oxidation, TPC, LAB, and total coliforms; increased a* value and residual nitrite (1%)	[54]
Chard and beetroot	Dry-cured traditional Spanish chorizo	6000 ppm (3000 ppm from each powder extract); *Pediococcus*, *Staphylococcus xylosus*, and *Staphylococcus carnosus* as starter culture	Ripening at 22 °C with 90% RH for 2 days and 14 °C with 70% RH for 23 days	n.e.	Reduced residual nitrate and nitrite, L*, a *, and b* values, hardness, and scores for redness, rancidity odor, acid flavor, rancidity flavor, and hardness; no effect on pH and protein oxidation; increased aw and sensory scores for brownness, general odor, cured odor, general flavor, cohesiveness, juiciness, and general acceptability	[55]
Chard and beetroot	Dry-cured traditional Spanish chorizo	6000 ppm (3000 ppm from each powder extract); *Pediococcus*, *Staphylococcus xylosus*, and *Staphylococcus carnosus* as starter culture	Stored at 4 °C for 125 days	n.e.	Reduced L*, a *, and b* values and hexanal and nonanal formation; no effect on pH and protein oxidation; increased aw	[55]
Beetroot	Fermented beef sausage	0.12%, 0.24%, and 0.35% powder; *Staphylococcus carnosus*, *Pediococcus acidilactici*, and *Lactobacillus sakei* as starter culture	Stored at 4 °C for 84 days	1.2–3.0 mg/kg	Similar pH, residual nitrite levels, TBARS, LAB (0.12% and 0.24%), L* and b* values, texture, and sensory attributes as controls with nitrite; increased a* value	[56]
Beetroot with celery or spinach powder	Fermented pork sausage	3 g/kg mixed extract; *Staphylococcus carnosus*, *Staphylococcus xylosus*, and *Lactobacillus sakei* as starter culture	During processing	b.d.l.	No effect on pH, LAB, aw, and sensory attributes	[57]

aw: water activity; LAB: lactic acid bacteria; RH: relative humidity; TBARS: thiobarbituric-acid-reactive substances; TPC: total plate count; VBN: volatile basic nitrogen; n.e.: not evaluated; b.d.l.: below detection limit.

### 3.3. Nitrate-/Nitrite-Rich Extracts from Beta vulgaris in Meat Products

The second strategy (combing the nitrate-rich extract with a starter culture) was explored in a recent study carried out by Ozaki et al. [54]. These authors evaluated the effect of beetroot extracts (0.5% and 1%) combined with *Staphylococcus carnosus* as a starter culture during processing and storage. The incorporation of both levels of extracts caused a significant increase in a* values as well as the formation of nitroso pigments in relation to controls with synthetic nitrite during processing. Other relevant outcomes were the reduction in lipid oxidation and L* and b* values during processing. In relation to the storage period, the sausages produced with beetroot extracts displayed higher a* values but with a significant decay in the content of nitroso pigments and L* and b* values during 60 days at 5 °C.

Martínez-Zamora et al. [55] evaluated the influence of combined chard and beetroot powders with a mix of starter cultures (*Pediococcus*, *Staphylococcus xylosus*, and *Staphylococcus carnosus*) in the processing and refrigerated storage of Spanish chorizo. Regarding the effect during processing, no effects on protein oxidation and pH were reported by the authors. Another important outcome was the modification of color. Both instrumental and sensory analyses indicated that samples produced with the natural extract reduce the intensity of lightness, redness, and yellowness. The samples produced with the combined extract received lower scores for rancidity odor, acid flavor, rancidity flavor, and hardness than control sausages. Additionally, the samples produced with chard and beetroot powders received higher scores for brownness, general odor, cured odor, general flavor, cohesiveness, juiciness, and general acceptability than control sausages. A similar outcome between these two treatments was reported during vacuum-packaged storage in terms of reduced instrumental color and the formation of volatile oxidation products, as well as for the lack of a significant effect on pH and protein oxidation.

A related study investigated the effect of beetroot extracts with a starter culture composed of *Staphylococcus carnosus*, *Pediococcus acidilactici*, and *Lactobacillus sakei* in fermented beef sausage [56]. In this product, the inclusion of beetroot extracts at different levels (0.12%, 0.24%, and 0.35%) provided a* values higher than those obtained from sausages with synthetic nitrite throughout the storage period. Moreover, these treatments with natural extracts provided similar results for pH, lipid oxidation, L* and b* values, texture, and sensory analysis in relation to sausages produced with nitrite salt. In terms of combination with other natural extracts, the use of beetroot extracts with powdered celery or spinach did not affect the pH, LAB, water activity, or sensory attributes of fermented sausages [57].

## 4. Nitrate and Residual Nitrite Content in Meat Products

The concern about the consumption of nitrite in meat products is related to the accumulation or formation of nitrosamines [58]. Essentially, these compounds are formed from the reaction between nitric oxide and secondary amines that can be favored by increasing temperature, low pH, increasing residual nitrite content, and increasing storage period [59]. The most common and widely study strategy, regardless of the meat product, is aiming at the reduction in residual nitrite at the end of processing [3].

An important outcome from the use of *Beta vulgaris* extracts as natural curing additives in meat products is the low residual nitrite content at the end of processing and throughout the storage period. This outcome was reported in studies using both pre-fermented extracts and combining nitrate-rich extracts with starter cultures with nitrate reductase activity [50,51,53,54,56]. It is important to mention that this effect is influenced by the concentration of extract used in the meat product, as observed in the studies carried out by Ozaki et al. [54] and Sucu and Turp [56]. Anyway, this outcome is an important health-related advance to obtain cured meat products with *Beta vulgaris* extracts.

## 5. Conclusions

*Beta vulgaris* varieties, especially chard and beetroot, are interesting sources of nitrate that can be explored in the production of cured meat products. Due to the variations in the nitrate content, it seems reasonable to recommend that the use of extracts from *Beta vulgaris* varieties has a quantification step (prior to use in meat products) in order to adjust the amount/volume of extract in the target concentration. Considering the proposed strategies using *Beta vulgaris* extracts as natural curing additives, it seems reasonable to indicate that the combination of these natural extracts with starter cultures is the most relevant strategy to obtain clean-label cured meat products. Further experiments should aim to increase the knowledge about the impact on the safety of muscle foods produced with natural extracts from *Beta vulgaris* varieties (especially against spore germination and toxin production by *Clostridium botulinum*), in addition to the quality attributes of meat products, and to explore the use of these extracts in reformulated meat products by reducing and/or replacing sodium chloride and animal fat to obtain healthier and functional meat products.

## Figures and Tables

**Table 1:** Nitrate content in *Beta vulgaris* varieties.

Scientific Name	Common Name	Plant Part (Nitrate Content in FW)	Class ^1^	Ref.
*Beta vulgaris* subsp. *vulgaris* var. *cicla*	Chard	Leaf (163–361 mg/kg)	VL–L	[25]
*Beta vulgaris* subsp. *vulgaris* var. *cicla*	Chard	Leaf (163–333 mg/kg)	VL–L	[24]
*Beta vulgaris* subsp. *vulgaris* var. *cicla*	Chard	Leaf (20–2820 mg/kg)	VL–VH	[23]
*Beta vulgaris* subsp. *vulgaris* var. *cicla*	Chard	Leaf (0–4362 mg/kg)	VL–VH	[22]
*Beta vulgaris* subsp. *vulgaris* var. *cicla*	Chard	Leaf (0–3509 mg/kg)	VL–VH	[21]
*Beta vulgaris* subsp. *vulgaris* var. *cicla*	Chard	Leaf (143–3050 mg/kg)	VL–VH	[33]
*Beta vulgaris* subsp. *vulgaris* var. *cicla*	Chard	Leaf (591–3571 mg/kg)	L–VH	[32]
*Beta vulgaris* subsp. *vulgaris* var. *cicla*	Chard	Leaf (261–5568 mg/kg)	L–EH	[31]
*Beta vulgaris* subsp. *vulgaris* var. *cicla*	Chard	Blade (353–662 mg/kg)	L–M	[30]
*Beta vulgaris* subsp. *vulgaris* var. *cicla*	Chard	Petiole (670–1022 mg/kg)	M–H	[30]
*Beta vulgaris* subsp. *vulgaris* var. *cicla*	Chard	Leaf (967–9093 mg/kg)	M–EH	[29]
*Beta vulgaris* subsp. *vulgaris* var. *cicla*	Chard	Leaf (1061 mg/kg)	H	[28]
*Beta vulgaris* subsp. *vulgaris* var. *cicla*	Chard	Leaf (2400 mg/kg)	H	[27]
*Beta vulgaris* L. spp. *cicla* cv. Seiyou Shirokuki	Chard	Leaf (1000–3000 mg/kg)	H–VH	[26]
*Beta vulgaris* subsp. *vulgaris* var. *cicla*	Chard	Leaf (1400–3400 mg/kg)	H–VH	[20]
*Beta vulgaris* subsp. *vulgaris* var. *cicla*	Chard	Leaf (3490–5912 mg/kg)	VH–EH	[19]
*Beta vulgaris* subsp. *vulgaris* var. *conditiva* Alef.	Beetroot	Leaf lamina (8–156 mg/kg)	VL	[34]
*Beta vulgaris* subsp. *vulgaris* var. *vulgaris*	Beetroot	Root (101–552 mg/kg)	VL–L	[35]
*Beta vulgaris* subsp. *vulgaris* var. *vulgaris*	Beetroot	Root (39–601 mg/kg)	VL–M	[36]
*Beta vulgaris* subsp. *vulgaris* var. *conditiva* Alef.	Beetroot	Leaf petiole (204–2496 mg/kg)	VL–VH	[34]
*Beta vulgaris* L. ssp. *esculenta* GURKE var. *rubra* L.	Beetroot	Root (700–850 mg/kg)	M	[37]
*Beta vulgaris* subsp. *vulgaris* var. *conditiva alef*.	Beetroot	Root (555–2896 mg/kg)	M–VH	[34]
*Beta vulgaris* subsp. *vulgaris* var. *conditiva*	Beetroot	Root (564–4626 mg/kg)	M–VH	[38]
*Beta vulgaris* subsp. *vulgaris* var. *vulgaris*	Beetroot	Root (1977 mg/kg)	H	[39]
*Beta vulgaris* L. subsp. *vulgaris* var. *conditiva alef*., mid–late variety, intensely purple, spherical, napiform	Beetroot	Root (2320 mg/kg)	H	[40]
*Beta vulgaris* var. *bengalensis*	Spinach beet	Leaf (268–811 mg/kg)	L–M	[41]
*Beta vulgaris* var. *bengalensis*	Spinach beet	Leaf (1801–2136 mg/kg)	H	[42]
*Beta vulgaris* (L.) subsp. *maritima* (L.) Arcang.	Sea beet	Leaves + young stems (673 mg/kg)	M	[43]

^1^ Classification of vegetables according to nitrate content [44]: very low (VL; <200 mg/kg); low (L; 200–500 mg/kg); medium (M; 500–1000 mg/kg); high (H; 1000–2500 mg/kg); very high (VH; 2500–5000 mg/kg); and extremely high (EH; >5000 mg/kg). All values are expressed in fresh weight (FW).

## Data Availability

Not applicable.
